# Decreasing Wellbeing and Increasing Use of Negative Coping Strategies: The Effect of the COVID-19 Pandemic on the UK Health and Social Care Workforce

**DOI:** 10.3390/epidemiologia3010003

**Published:** 2022-01-18

**Authors:** Patricia Gillen, Ruth D. Neill, Jill Manthorpe, John Mallett, Heike Schroder, Patricia Nicholl, Denise Currie, John Moriarty, Jermaine Ravalier, Susan McGrory, Paula McFadden

**Affiliations:** 1 School of Nursing, Jordanstown Campus, Ulster University, Shore Road, Newtownabbey BT37 0QB, UK; s.mcgrory@ulster.ac.uk; 2 Southern Health and Social Care Trust, 10 Moyallen Road, Gilford BT63 5JX, UK; p.mcfadden@ulster.ac.uk; 3 School of Applied Social Policy Sciences, Magee Campus, Ulster University, Londonderry BT48 7JL, UK; r.neill@ulster.ac.uk (R.D.N.); p.nicholl2@ulster.ac.uk (P.N.); 4 NIHR Health and Social Care Workforce Research Unit, King’s College London, 22 Kingsway, London WC2B 6LE, UK; jill.manthorpe@kcl.ac.uk; 5 School of Psychology, Coleraine Campus, Ulster University, Cromore Road, Coleraine BT52 1SA, UK; j.mallett@ulster.ac.uk; 6 Queen’s Management School, Queen’s University Belfast, Riddel Hall, 185 Stranmillis Road, Belfast BT9 5EE, UK; h.schroder@qub.ac.uk (H.S.); d.currie@qub.ac.uk (D.C.); 7 School of Social Sciences, Education and Social Work, Queen’s University Belfast, 69–71 University Street, Belfast BT7 1HL, UK; j.moriarty@qub.ac.uk; 8 School of Science, Bath Spa University, Newton Park, Newton St. Loe, Bath BA2 9BN, UK; j.ravalier@bathspa.ac.uk

**Keywords:** COVID-19, healthcare workforce, social care workforce, social work, United Kingdom, coping, wellbeing, quality of working life, survey

## Abstract

Many health and social care (HSC) professionals have faced overwhelming pressures throughout the COVID-19 pandemic. As the current situation is constantly changing, and some restrictions across the UK countries such as social distancing and mask wearing in this period (May–July 2021) began to ease, it is important to examine how this workforce has been affected and how employers can help rebuild their services. The aim of this study was to compare cross-sectional data collected from the HSC workforce in the UK at three time points during the COVID-19 pandemic: Phase 1 (May–July 2020), Phase 2 (November 2020–January 2021) and Phase 3 (May–July 2021). Respondents surveyed across the UK (England, Wales, Scotland, Northern Ireland) consisted of nurses, midwives, allied health professionals, social care workers and social workers. Wellbeing and work-related quality of life significantly declined from Phase 1 to 3 (*p* < 0.001); however, no significant difference occurred between Phases 2 and 3 (*p* > 0.05). Respondents increasingly used negative coping strategies between Phase 1 (May–July 2020) and Phase 3 (May–July 2021), suggesting that the HSC workforce has been negatively impacted by the pandemic. These results have the potential to inform HSC employers’ policies, practices, and interventions as the workforce continues to respond to the COVID-19 virus and its legacy.

## 1. Introduction

Declared a pandemic in March 2020 [1], the novel Coronavirus (COVID-19) outbreak spread rapidly worldwide, changing how individuals lived and worked, with a severe impact on health, education, politics, the environment, and the economy [2,3,4]. According to the World Health Organisation dashboard [5], over 5.4 million deaths and 281.8 million cases have occurred worldwide so far, with 148,021 deaths and 12.3 million cases in the United Kingdom (UK) as of 4 January 2022. The pandemic initially introduced severe restrictions on daily activity and travel. Guidance on social distancing, mask wearing, working from home if possible and the importance of good hygiene practices, such as handwashing were highlighted in public health campaigns globally [6,7,8,9,10]. These changes affected working conditions for millions, particularly those working in the health and social care (HSC) sector, many of whom also experienced new pressures at work.

Accounting for over 3 million people across the UK [11], health and social care (HSC) workers provide essential long term and short term care to individuals and families across a range of populations (adults, children, learning disability, mental health, physical disability, older people’s care, and so on) and settings. How direct care and support are provided has altered much during the COVID-19 pandemic. Pre-pandemic, the HSC workforce was already viewed as a high risk group for mental health problems, severe stress, lower wellbeing and higher burnout as budget cuts, lack of resources and policy reforms placed services under substantial pressures taking a detrimental toll on HSC staff and employers [12,13,14,15]. The current HSC workforce faced long-lasting consequences due to the heavy responsibility placed on these professionals during the pandemic [16,17,18,19]. Even when restrictions ease in many countries, with removal of the full social distancing and mask-wearing measures in most environments, the burden on this workforce remains. Whilst considerable measures such as COVID-19 and flu vaccination programmes, promotion of maskwearing indoors and good hygiene have been widely promulgated and successful across the UK [20,21,22], the virus spread is still present, particularly as the seasons change and new virus variants circulate.

Emerging research during the COVID-19 crisis documented the negative toll of working in the HSC sector, with adverse consequences in terms of coping strategies adopted, lower work-related quality of life and wellbeing for HSC employees. The pandemic changed how care was delivered, with staff often working under greater pressure, straining their current coping strategies as job demands and stress increase [12,16,23,24]. Isolation, social restrictions, changes in care and operational pathways, communication problems between employees, employers and providers, longer waiting lists, and new working practices have been further exacerbated by the stress of the pandemic [12,25,26,27]. Changes in working conditions and lifestyles have led to burnout amongst some, with higher job demands, fewer available resources and staff turnover/shortages affecting mental wellbeing and quality of working life [16,17,24,25,28,29,30,31]. This evidence suggests a need for continued exploration of burnout, coping strategies, mental wellbeing, and work-related quality of life in the HSC sector over the course of the pandemic and beyond.

This current study aimed to compare cross-sectional data collected from nurses, midwives, AHPs, social care workers and social workers in the UK at three different time points (Phase 1: May–July 2020; Phase 2: Nov 2020–Jan 2021; Phase 3: May–July 2021) during the COVID-19 pandemic. It specifically examined self-reported coping strategies, mental wellbeing, burnout, and quality of working life. This paper aims to provide an insight into the wellbeing of nurses, midwives, AHPs, social care workers and social workers, and explores the importance and relevance of certain variables between all three different phases of the COVID-19 pandemic. Examining the difference in wellbeing and work-related quality of life over the course of the pandemic is crucial in helping to identify any relevant supports for this workforce. The results of Phase 3 of this study will be used to expand on recommendations from earlier phases that could be used to inform policies and procedures for HSC employers to help counteract the adverse long-term effects the pandemic on mental wellbeing and work-related quality of life. Other papers linked to the wider study have explored the findings of the earlier phases [32,33], social care and social workers [34] and the ‘clapping for carers’ initiative [35].

## 2. Materials and Methods

### 2.1. Study Design and Sample

This study forms a part of a continuous research programme entitled ‘Health and social care workers’ quality of working life and coping while working during the COVID-19 pandemic’. The overall research project focuses on mental wellbeing, quality of working life, burnout and coping strategies in nurses, midwives, allied health professionals (AHPS), social care workers and social workers from across the UK who are employed in a range of settings such as the community, day services, care homes and hospitals. The findings from this research aim to provide evidence-based recommendations for supporting the HSC workforce not just during the COVID-19 crisis, but also during business-as-usual times. The wider study uses a cross-sectional design, and the data for the current study presented here were collected at three time points: Phase 1: 7 May to 3 July 2020, Phase 2: 17 November 2020 to 1 February 2021 and Phase 3: 10 May to 2 July 2021. The research used an online survey with previously validated measures that also contained a small number of qualitative questions to further understand the self-reported experience of HSC professionals aged from 16 years upwards. The survey draws on a convenience sample of those who choose to participate voluntarily by accessing the survey through social media platforms such as Facebook and Twitter, professional associations, workplace unions, professional communications, employers, and regulatory bodies. An electronic link and QR code to the Participant Information Sheets, consent and survey were provided through these platforms to try and reach a wider population of health and social care workers across the UK. Participants could withdraw from the study at any time by not completing the survey. Clicking on the arrow to proceed after the Participant Information Sheet and completion of the survey indicated consent. Study eligibility was based on respondents self-reporting their occupation and country of work. For all phases, data were collected anonymously through the Qualtrics platform which hosted the anonymous online survey tool. Phase 1 received a total of 3290 responses, Phase 2 received 3499 responses and Phase 3 received 2721 responses. Demographic and work-related characteristics of the sample by study phase are presented in Table 1.

### 2.2. Ethical Considerations

Ethical approval was obtained from the Research Ethics Filter Committee of the School of Nursing at Ulster university (Ref No: 2020/5/3.1, 23 April 2020, Ulster University, IRAS Ref No. 20/0073) phases of the study and Trust Governance approval (for Northern Ireland only) was gained from Health and Social Care Trusts from Phase 2 onwards. This allowed the link to the questionnaire in Phase 2 and Phase 3 to be shared with HSC staff in Northern Ireland via their Trust work emails. While respondents were volunteering to undertake the anonymous survey, it was thought possible that during completion that they may become distressed. Therefore, at both the bottom of the Participant Information Sheet and the end of the survey, respondents were provided with information about whom to contact if they needed support. Permission for the use of the scales used in the questionnaire was provided by original authors, and consent and confidentiality were addressed in Participant Information Sheets provided at the start of the survey.

### 2.3. Measures

#### 2.3.1. Demographics and Work-Related Characteristics

Respondents were asked about their demographic and work-related characteristics. The variables that were consistent across all three time points of the wider study and relevant to the analysis in this present paper are sex, age, country of work, ethnicity, occupation, years of work experience, disability status and redeployment status during the pandemic.

#### 2.3.2. Mental Wellbeing

Mental wellbeing was assessed by the 7 item, short version of the Warwick-Edinburg Mental Wellbeing Scale (SWEMWS) which has statements about thoughts and feelings [36]. The scale is scored by summing the scores for each item, which range from 1 (None of the time) to 5 (All of the time), then the total raw scores are transformed by the SWEMWS conversion table into metric scores. Higher scores indicate positive wellbeing with scores ranging from 7 to 35. The scale has shown good psychometric properties within the UK general population [37] and Danish adult populations [38]; in the present study, internal consistency was good in the final sample (α = 0.87).

#### 2.3.3. Work-Related Quality of Life

Overall, work-related quality of life was assessed with the 23-item Work Related Quality of Life (WRQoL) scale [39] which reports employees’ perceived quality of life measured through 6 sub domains (job career satisfaction, stress at work, working conditions, control at work, general wellbeing, and home-work interface). Respondents were asked to rate the items using a five-point Likert scale ranging from 1 (Strongly disagree) to 5 (Strongly agree) to indicate their attitudes to the factors that influence their quality of working life with higher scores indicating better work-related quality of life. The scale has previously shown good psychometric properties [39,40] and in this present research internal consistency was good in the final sample (α = 0.88).

#### 2.3.4. Burnout

Burnout was assessed using the 19-item Copenhagen Burnout Inventory (CBI) [41], which measured three different areas of burnout: personal, work-related, and client-related. Twelve items were rated on a five-point Likert scale ranging from 0 to 100 (never/almost never to always) while seven items offered response categories ranging from a very low degree to very high degree. Scale scores were computed as the average (mean) scores across all relevant items. For each area of burnout, a mean score was calculated, with higher scores indicating a higher level of possible burnout. In the current study, the burnout scores in each area were categorised into low, moderate, high, and severe burnout using the cut-off scores as frequently cited in the literature [42]. The scale has shown good psychometric properties, for example, personal (α = 0.89), work-related (α = 0.90) and client-related (α = 0.90) burnout [43] and, in this present study, internal consistency was good in the final sample for personal burnout (α = 0.91), work-related burnout (α = 0.79) and client-related burnout (α = 0.87). Burnout was assessed in Phases 2 and 3 only.

#### 2.3.5. Coping

Coping was assessed by two scales, the Strategies for Coping with Work and Family Stressors Scale [44] and the Brief COPE scale [45]. Only 20 items from the Brief COPE scale [45] were used to assess ten different coping strategies (active coping, planning, acceptance, positive reframing, use of emotional support, use of instrumental support, venting, substance use, behavioural disengagement, and self-blame). The scale assesses the frequency with which the respondent has been using the strategies described in the items using a four-point Likert scale ranging (1 = ‘I haven’t been doing this at all’ to 4 = ‘I’ve been doing this a lot’). The Cronbach’s alpha for the 20-item scale was acceptable in this present study (α = 0.82).

The 15 items from Clark et al.’s [44] scale assessed five different coping strategies (family–work segmentation, work-family segmentation, working to improve skills/efficiency, recreation and relaxation and exercise). Respondents indicated how often they had been doing what was derived by the statements on the six-point Likert scale (1 = ‘Never have done this’ to 6 = ‘Almost always do this’) with a mean score for each domain computed. The Cronbach’s alphas for the scale were acceptable in all phases in this present (α = 0.83).

### 2.4. Data Analysis

All three datasets from the respective phases were recoded and merged into SPSS. Data analyses were conducted using SPSS 26 and any missing data were addressed prior to beginning the analysis. Respondents who did not complete all items on one or more of the scales (SWEMWBS, WRQoL, Brief COPE, and Clark’s coping) were excluded from the merged dataset for all three phases (*n* = 2008). Those who indicated gender as neither or missing (*n* = 26 = 0.3%) were excluded as the subgroup sample was so low. The remaining missing data on the variables relevant to the analyses were minimal (0.22%). The SWEMWBS, WRQoL, Burnout and the coping items were treated as continuous variables and missing data on these items were estimated using the EM algorithm for single imputation in SPSS. Missing values on the demographic and work-related variables were minimal (0.04%) and they were not estimated. Instead, listwise deletion was used in the analyses.

Preliminary analyses using descriptive statistics were conducted to provide basic information regarding the variables included in the study. Multivariate statistics using Mann–Whitney U and Kruskal–Wallis tests were used to examine the difference in the outcome variables, across the various scales for burnout, wellbeing, WRQoL and coping strategies; across the three study phases. To account for the different distribution of occupational groups and countries across the three study phases, descriptive statistics for the outcome variables (wellbeing, quality of working life, burnout, and coping strategies) were weighted by occupation and country.

## 3. Results

### 3.1. Sample Characteristics

The final sample across all three phases consisted of 7476 HSC respondents (Phase 1: *n* = 2546; Phase 2: *n* = 2718; Phase 3: *n* = 2212). Respondents were mainly white (95.5%), predominately female (87.9%), just under half had between 11 and 30 years’ experience (49.7%), one-third were in the 50–59 age category (31.4%), and nearly half of the respondents were from Northern Ireland (42.5%). Over two-thirds of the sample were working in Social Care and Social Work (68.9%). Just under half of the respondents worked in the community (49.8%), with main areas of practice being with older people and other adults (43.3%). Over half of the respondents were married (52.9%), reported no disability (87.2%) and had not been redeployed during the COVID-19 pandemic (86.9%). A full breakdown of the demographics is reported in Table 1.

### 3.2. Descriptive Statistics

Descriptive statistics of the outcome variables for each phase are shown in Table 2 and Table 3. The results showed that respondents’ wellbeing and work-related quality of life decreased across each phase. Respondents appeared to be using several positive coping strategies less frequently (active coping, positive reframing, acceptance, use of emotional support and use of instrumental support, work-family segmentation, working to improve skills/efficiency, recreation and relaxation and exercise). However, many of the negative coping strategies were more frequently used as the phases of the study progressed (substance use, behavioural disengagement, and self-blame). All domains of burnout showed increased personal, work-related, and client-related burnout between Phases 2 and 3.

### 3.3. Mulitvariate Analysis

Data were not normally distributed and therefore Kruskal–Wallis H tests were used to compare the scores on wellbeing, work-related quality of life, and the domains of coping. As burnout was only measured in Phases 2 and 3, Mann-Whitney U tests were performed for each domain. The results showed that wellbeing and overall WRQoL significantly differed across the occupation groups in Phases 1, 2 and 3 (*p* < 0.05). The results showed that wellbeing (H (2) = 112.45, *p* < 0.001) significantly declined across the Phases. Phase 1 respondents reported the highest wellbeing (20.96), compared to Phase 2 (20.21) and Phase 3 (20.18). Dunn’s pairwise comparison (adjusted using Bonferroni correction) revealed a significant difference between wellbeing in Phase 1 and 3 (*p* < 0.001) while no significant difference between Phases 2 and 3 (*p* = 0.099) was found. Overall WRQoL (H (2) = 305.52, *p* < 0.001) significantly decreased across the three phases, with a mean rank wellbeing score of 78.09 for Phase 1, 72.53 for Phase 2 and 71.98 for Phase 3. Dunn’s pairwise comparison revealed a significant difference in overall WRQoL between Phases 1 and 3 (*p* < 0.001) with no significant difference between Phases 2 and 3 (*p* = 0.355).

Active coping (H(2) = 255.71, *p* < 0.001), positive reframing (H(2) = 125.1, *p* < 0.001), acceptance (H(2) = 140.72, *p* < 0.001), use of emotional support, (H(2) = 30.64, *p* < 0.001), use of instrumental support (H(2) = 23.73, *p* < 0.001), work–family segmentation (H(2) = 50.03, *p* < 0.001), working to improve skills/efficiency (H(2) = 139.37, *p* < 0.001), recreation and relaxation (H(2) = 82.89, *p* < 0.001), exercise (H(2) = 317.67, *p* < 0.001) and venting (H(2) = 281.17, *p* < 0.001) all significantly decreased across the phases. Planning (H (2) = 49.38, *p* < 0.001), Self-blame (H (2) = 353.94, *p* < 0.001), Substance use (H (2) = 40.04, *p* < 0.001) and behavioural disengagement (H (2) = 201.61, *p* < 0.001) were coping strategies which all significantly increased across the phases. Family–work segmentation was the only coping strategy to exhibit no significant difference across the phases (H (2) = 0.673, *p* = 0.793).

Mann–Whitney U tests showed that personal burnout (U = 2947950.5, *p* < 0.001, r = 0.08), work-related burnout (U = 3095879.5, *p* < 0.001, r = 0.13), and client-related burnout (U = 2794055.5, *p* = 0.02, r = 0.04), were all significantly higher in Phase 3 compared to Phase 2. Personal burnout significantly increased between Phase 2 and 3 for AHPs U = 73636.5, *p* < 0.01), social care workers (U = 59361, *p* < 0.001) and social workers (U = 670174.5, *p* < 0.001). Work-related burnout significantly increased between Phase 2 and 3 for social care workers (U = 61551, *p* < 0.001) and social workers (U = 659191.5, *p* < 0.001). For client-related burnout only social care workers had a significantly higher score between the Phases 2 and 3 (U = 609925.500, *p* < 0.05). Overall midwives had the lowest wellbeing and WRQoL scores but also exhibited the highest levels of personal, work-related and client burnout. For the overall sample, there was an increase in high-severe personal burnout from 27.6% in Phase 2, to 36.5% in Phase 3; high-severe work-related burnout increased from 20.8% to 26%, while high-severe client-related burnout increased from 2.0% to 3.2%, respectively.

## 4. Discussion

### 4.1. Main Messages in Comparison with the Literature

The aim of the present paper is to compare cross-sectional data collected from nurses, midwives, AHPs, social care workers and social workers in the UK at three different time points (Phase 1: May–July 2020; Phase 2: Nov 2020–Jan 2021; Phase 3: May–July 2021) during the COVID-19 pandemic. The results showed that, at all time points, average mental wellbeing scores in this study are nearly three points lower than previously reported, as the population Norm has been reported as a mean score of 23.6 [37,46]. These findings are also lower than previous UK findings pre-pandemic in which a mean score of 25.2 (3.1) was recorded in community nurses [47]. While similarities during the pandemic have been reported by Sumner & Kinsella [48] amongst UK and Ireland HSC respondents who recorded a mean SWEWBS score of 22.7 (4.91); Smith et al. [49] reported a mean SWEWBS score of 20.8 (5.1) in UK based respondents. A study by Dawson and Goligani-Moghaddam [50] also found a mean SWEWBS score of 20.21 (3.97) across the general UK population, suggesting that both HSC workers and the general public have been severely affected in terms of their wellbeing due to the ongoing pandemic. This suggests that the pandemic may potentially influence the wellbeing of the HSC workforce due to the increased demands and changes within their personal life due to COVID-19-related problems and stressors.

In this current analysis, WRQoL scores decreased from a mean score of 78.09 (17.50) in Phase 1 to 71.98 (15.88) in Phase 3. With these phases conducted a year apart, these results indicate a significant decrease in WRQoL scores. Social Workers, Social Care workers and midwives continued to show a decline in WRQoL scores over Phases 1, 2 and 3; this could be a result of the change to their working conditions, with many of these respondents experiencing restrictions to their jobs or having to work from home. The findings from Phase 1 are comparable to the mean normative score of 3.44 (78.09/23 = 3.40) derived from the UK NHS workforce study [51]; however, as the pandemic continued, results in Phase 2 (72.53/23 = 3.15) and Phase 3 (71.98/23 = 3.13) were considerably lower. Moreover, the findings of this study also coincide with average quality of working life reported by Easton et al. [51]. Mendes and Pereira [52] reported a value of 3.40 in the general Portuguese working population, suggesting that healthcare workers may have lower WRQoL. Pre-pandemic, within the AHPs, one study reported WRQoL scores of 82.92 (14.17) for occupational therapists in Iran [53] while another study of Vietnam’s healthcare professionals reported higher scores of 95.52 and 92.10 during the pandemic [54], suggesting that these professionals had higher WRQoL than UK-based respondents. However, during the pandemic, lower scores have been reported amongst nurses in hospitals in Iran [55,56], suggesting that country of work and different working practices could influence total WRQoL.

Burnout (measured in Phases 2 and 3 only) was found to significantly increase during the pandemic for the respondents surveyed. Work-related burnout was much higher in this study (Phase 2—56.33 and Phase 3—60.32) than reported among NHS staff in the UK earlier in the pandemic, with Gemine et al. [57] stating work-related burnout to be 45.7 (15.7), suggesting that redeployment and changing advice and conditions may result in increased burnout; a similar rationale may be attributed to this study. Additionally, the findings in this present study are higher than previous findings from healthcare workers in India [58] in which personal burnout was reported as 49.72 (18.68) and work-related burnout as 39.39 (20.43). A study of healthcare workers in Portugal measured burnout as low or high; high personal burnout was reported at 52.5%, high work-related burnout 53.1% and 35.4% had high client-related burnout [59]. In comparison with this present study (with moderate–severe categories combined for comparison), HSC workers in the UK experienced higher levels of high personal (74.2%) and work-related burnout (66.0%) but lower client-related burnout (20.1%).

As the pandemic continued, our results indicate that more negative avoidant coping strategies (substance use, behavioural disengagement and self-blame) were being adopted by the HSC workforce and less use of more positive approach type strategies (active coping, positive reframing, acceptance, use of emotional support, use of instrumental support). Similarities in the mean scores over the three phases can be found with a longitudinal study in Belgium by Van Steenkiste et al. [60], with the suggestion that avoidance strategies worsen from the first time point as the pandemic continued, though these authors reported no significant difference in coping over time in Belgian healthcare workers, whereas this current study found a significant difference. A study by Cansiz et al. [61] examined coping among Turkish healthcare professionals reporting that frontline workers used more acceptance and active coping strategies than non-frontline workers during the pandemic. In comparison with this present study, Cansiz et al. [61] reported higher use of negative strategies, such as self-blame, substance use, emotional support and behavioural disengagement, but also higher use of planning and emotional support strategies. Studies from Canestrari et al. [62] in Italy and Vera-Monge et al. [63] in Spain reported lower scores for the usage of positive approach strategies and negative avoidant strategies, while a study involving Greek healthcare professionals reported higher scores for the usage of positive approach strategies and lower usage of avoidant strategies than this present study [64].

Engaging in the negative, more avoidant coping strategies was also found in our earlier study phases [32,33,34] and within the literature, with the usage of these strategies being found to have a negative relationship and impact on mental wellbeing and resulting in increased burnout within the HSC workforce [50,60,62,63,65,66,67]. Amongst respondents in Phase 3 of this present study there was widespread recognition that pandemic-related work responsibilities had affected respondents’ quality of home life, with working from home not enabling a break from work for many of this workforce and it was noted that an emphasis should be placed on re/creating a more healthy work–life balance [68]. Overall, evidence suggests that the use of positive coping strategies are essential in reducing stressors, improving wellbeing and work-related quality of life which can then reduce burnout [64,69,70,71].

### 4.2. Limitations and Strengths

This study has several limitations. While this study highlights any statistically significant associations between the examined outcomes, the study design for each phase of the research was cross-sectional, thus only providing a snapshot of the outcomes at that specific point of time. In cross-sectional studies it is impossible to infer causality which limits evaluation of associations between outcomes [72,73]. However, a benefit of repeated cross-sectional studies is that they can provide information over time to inform public health planning and policies, which is important in the sustainability of the HSC workforce. Additionally, a strength of this study is the time periods in which the data were collected as this represents different levels of pressures for the workforce during the pandemic. The survey is self-report therefore may be subject to recall bias or social desirability bias [74,75], although the surveys were anonymous. Another limitation is that a majority of respondents were from the social care and social work sectors (68.9%) and therefore not representative of the whole health and social care sector. This may have been the result of other workforce surveys in the healthcare sector/NHS which were directed at nurses, midwives and AHPs. As noted previously, data were weighted to account for the different distribution of occupational groups and countries across the three study phases, which diminishes the effects of inherent biases of the survey, addresses sampling and none response bias, while allowing for a more accurate representation of the population under examination [76,77].

Data were collected online using a convenience sampling method through extensive sharing of a survey link through social media platforms. While this was an efficient, simple and pragmatic way to safely gather data from a large sample during the COVID-19 pandemic, it may increase the risk of selection bias in that the sample may be over-representative either of workers with sufficient time to complete a survey, or those concerned about the wellbeing of themselves and colleagues and therefore more motivated to register their views [74,78,79]. This therefore can result in a lack of clear generalisability within the results and increased risk of bias [80]. Additionally, recruiting a sample which ethnically resembles the workforce has proven a challenge and most of respondents within this study identify as white ethnicity (95.5%), which may be a result of the convenience sampling technique utilised. We did not assess any previous co-morbidities among respondents and the presence of these co-morbidities might have influenced how mental-wellbeing, work-related quality of life and coping were reported.

### 4.3. Implications

As the HSC sector begins to rebuild pre-pandemic services, several elements must be addressed to prevent further burden on this already stretched workforce. Protecting and managing the mental wellbeing and quality of working life of those working in HSC is necessary for long-term sustainability of the workforce and the services they provide. Managing fresh challenges, preparing for possible future epidemic/pandemics and providing ongoing support are crucial. There is still some uncertainty for the future deployment of this workforce therefore new or updated supports and policies need to be reviewed and implemented. It is important to manage coping strategies as there was significant decline in the number of positive strategies used from May 2020 to July 2021. Based on our good-practice recommendations [68] which emerged from the data, sustained support needs to occur across three system levels (individual, organisational and policy).

The pandemic has further highlighted problems within the HSC system and, as job demands increase and will continue to do so in the aftermath of COVID-19, communication will be important for employers to maintain and develop. Signposting staff to services for mental health and wellbeing will be important, while highlighting that staff are valued is essential for cohesion and job satisfaction. Furthermore, evidence also suggests that support may help mitigate some of the negative impacts of COVID-19 on HSC staff, particularly continued support from leaders and supervisors while being provided relevant and honest information about COVID-19 to respond to changing conditions of work [81,82]. The unprecedented pressure on HSC has shone a light on the persistent under-resourcing of staff and infrastructure. Therefore, concerted efforts are required to make work attractive and welcoming. Another implication of these findings is that wellbeing and work-related quality of life may improve with more flexibility in employment, through regular breaks and possible options to work from home. Flexibility in the workplace has been acknowledged as reducing the stressors around work–life balance which may in the long-term significantly improve mental health and quality of life as it can increase resilience [83,84,85].

## 5. Conclusions

In summary, our findings support other research which highlights that the COVID-19 pandemic has been overwhelming for the much of the HSC sector. While most of the HSC workforce has been resilient throughout this crisis, continuing changes to home–work life, job responsibilities and increased work-related pressures worsened by the COVID-19 pandemic could have a lasting impact on wellbeing, coping, burnout and work-related quality of life. With few studies assessing these outcomes across the HSC workforce, it is important for research to examine the long-term impact of the pandemic on the frontline of care and to compare each outcome across the different occupations

## Figures and Tables

**Table 1:** Demographics and work-related characteristics of sample (Phase 1: *n* = 2546; Phase 2: *n* = 2718; Phase 3: *n* = 2212).

Variable	Phase 1 (7 May–3 July 2020)	Phase 2 (17 November 2020–1 February 2021)	Phase 3 (10 May–2 July 2021)
Sex			
Female	2221 (87.2%)	2398 (88.2%)	1952 (88.2%)
Male	325 (12.8%)	320 (11.8%)	260 (11.8%)
Age			
16–29	303 (11.9%)	304 (11.2%)	185 (8.4%)
30–39	539 (21.2%)	628 (23.1%)	412 (18.6%)
40–49	753 (29.6%)	714 (26.3%)	598 (27.1%)
50–59	755 (29.7%)	811 (29.8%)	783 (35.4%)
60+	195 (7.7%)	261 (9.6%)	232 (10.5%)
Ethnic background			
White	2395 (94.2%)	2605 (96.1%)	2129 (96.4%)
Black	74 (2.9%)	40 (1.5%)	30 (1.4%)
Asian	29 (1.1%)	26 (1.0%)	19 (0.9%)
Mixed	44 (1.7%)	41 (1.5%)	31 (1.4%)
Country of work			
England	905 (35.5%)	633 (23.3%)	434 (19.5%)
Scotland	107 (4.2%)	350 (12.9%)	611 (27.6%)
Wales	146 (5.7%)	843 (31.0%)	266 (12.0%)
Northern Ireland	1388 (54.5%)	892 (32.8%)	901 (40.7%)
Occupational group			
Nursing	142 (5.6%)	283 (10.4%)	459 (20.8%)
Midwifery	139 (5.5%)	57 (2.1%)	136 (6.1%)
Allied Health Professionals	311 (12.2%)	490 (18.0%)	312 (14.1%)
Social Care Workers	919 (36.1%)	946 (34.8%)	674 (30.5%)
Social Workers	1035 (40.7%)	942 (34.7%)	631 (28.5%)
Number of years of work experience			
Less than 2 years	211 (8.3%)	183 (6.7%)	116 (5.2%)
2–5 years	373 (14.7%)	373 (13.7%)	282 (12.8%)
6–10 years	407 (16.0%)	449 (16.5%)	303 (13.7%)
11–20 years	688 (27.0%)	824 (30.3%)	596 (27.0%)
21–30 years	572 (22.5%)	548 (20.2%)	488 (22.1%)
More than 30 years	295 (11.6%)	340 (12.5%)	425 (19.2%)
Place of Work			
Hospital	250 (9.8%)	304 (11.2%)	458 (20.7%)
Community	1446 (56.9%)	1277 (47.1%)	989 (44.8%)
General Practice based	12 (0.5%)	45 (1.7%)	31 (1.4%)
Care Home	303 (11.9%)	267 (9.9%)	200 (9.1%)
Day Care	47 (1.8%)	92 (3.4%)	61 (2.8%)
Other	484 (19.0%)	725 (26.8%)	469 (21.2%)
Main area of practice			
Children	531 (20.9%)	714 (26.3%)	389 (17.6%)
Midwifery	138 (5.4%)	55 (2.0%)	137 (6.2%)
Adults	485 (19.1%)	594 (21.9%)	552 (25.0%)
Physical Disability	50 (2.0%)	51 (1.9%)	35 (1.6%)
Learning Disability	285 (11.2%)	295 (10.9%)	233 (10.5%)
Older People	602 (23.7%)	533 (19.6%)	470 (21.2%)
Mental Health	216 (8.5%)	274 (10.1%)	219 (9.9%)
Other	238 (9.4%)	202 (7.4%)	177 (8.0%)
Disability status			
Yes	222 (8.7%)	271 (87.7%)	285 (12.9%)
No	2268 (89.1%)	2384 (87.7%)	1865 (84.4%)
Unsure	56 (2.2%)	62 (2.3%)	61 (2.8%)
Redeployment			
Yes	361 (14.2%)	319 (11.7%)	299 (13.5%)
No	2185 (85.8%)	2399 (88.3%)	1913 (86.5%)

Note: Presented are column percentages, which are valid percentages to account for missing data.

**Table 2:** Means and standard deviations (in brackets) for the wellbeing, work-related quality of life (WRQoL) and Burnout by occupation and across phases.

Variables	Occupation				
	Social Work	Social Care	AHP	Midwifery	Nursing
Wellbeing					
Phase 1	21.32 (3.34)	20.90 (3.92)	21.32 (3.32)	20.87 (3.24)	21.15 (3.68)
Phase 2	20.11 (3.20)	20.05 (3.59)	20.60 (3.36)	19.81 (2.37)	20.43 (3.16)
Phase 3	19.83 (3.25)	19.82 (3.89)	20.72 (3.87)	19.28 (3.08)	20.59 (3.47)
WRQoL					
Phase 1	80.67 (13.58)	79.16 (15.62)	82.06 (12.42)	77.43 (15.28)	75.11 (18.64)
Phase 2	74.06 (15.17)	73.38 (16.35)	74.10 (16.11)	66.53 (15.95)	71.19 (14.43)
Phase 3	69.78 (15.97)	70.69 (15.73)	75.16 (18.34)	64.80 (12.83)	74.57 (15.30)
Personal Burnout					
Phase 2	62.86 (19.81)	54.17 (22.84)	57.00 (17.96)	66.76 (13.59)	62.74 (18.39)
Phase 3	62.86 (19.81)	59.65 (22.76)	62.01 (21.93)	72.13 (16.95)	61.42 (22.14)
Work-related Burnout					
Phase 2	60.45 (19.91)	59.74 (20.76)	54.77 (20.55)	66.21 (18.98)	57.55 (18.94)
Phase 3	64.36 (19.67)	63.67 (20.54)	55.22 (24.17)	70.55 (17.63)	57.42 (22.04)
Client-related Burnout					
Phase 2	30.63 (20.53)	25.49 (20.30)	28.17 (19.44)	31.02 (22.67)	28.21 (20.49)
Phase 3	32.71 (22.97)	27.48 (20.23)	30.34 (23.57)	34.27 (20.94)	27.77 (22.87)

**Table 3:** Means and standard deviations (in brackets) for wellbeing, work-related quality of life (WRQoL), coping strategies and burnout across study phases.

Variable	Phase 1 (*n* = 2546)	Phase 2 (*n* = 2718)	Phase 3 (*n* = 2212)	Phase Comparison
	M (SD)			*p*-Value
Wellbeing	20.96 (3.78)	20.21 (3.36)	20.18 (3.81)	<0.001
Quality of working life	78.09 (17.50)	72.53 (15.92)	71.98 (15.88)	<0.001
Coping strategies				
Active coping	6.00 (1.64)	5.47 (1.70)	5.32 (1.87)	<0.001
Planning	5.81 (1.81)	5.52 (1.86)	5.57 (1.74)	<0.001
Positive reframing	5.85 (1.65)	5.57 (1.70)	5.37 (1.69)	<0.001
Acceptance	6.39 (1.53)	6.18 (1.51)	5.97 (1.47)	<0.001
Use of emotional support	4.93 (1.77)	4.74 (1.83)	4.69 (1.76)	<0.001
Use of instrumental support	4.34 (1.83)	4.30 (1.79)	4.11 (1.74)	<0.001
Venting	3.51 (1.43)	4.15 (1.64)	4.07 (1.61)	<0.001
Substance use	2.75 (1.41)	2.81 (1.44)	2.98 (1.56)	<0.001
Behavioural disengagement	2.73 (1.25)	3.00 (1.37)	3.22 (1.53)	<0.001
Self-blame	3.42 (1.81)	4.01 (1.88)	4.24 (1.87)	<0.001
Family–work segmentation	5.13 (0.84)	5.12 (0.84)	5.13 (0.86)	0.673
Work–family segmentation	4.66 (1.05)	4.59 (1.07)	4.44 (1.25)	<0.001
Working to improve skills/efficiency	4.49 (1.09)	4.18 (1.15)	4.23 (1.12)	<0.001
Recreation and relaxation	3.75 (1.23)	3.55 (1.31)	3.46 (1.22)	<0.001
Exercise	3.97 (1.41)	3.65 (1.38)	3.34 (1.41)	<0.001
Burnout				
Personal	Not measured	61.21 (19.77)	63.60 (21.17)	<0.001
Work-related	Not measured	56.33 (21.10)	60.32 (21.72)	<0.001
Client-related	Not measured	27.95 (20.35)	29.74 (21.76)	<0.001

## Data Availability

The data presented in this study are available on request from the corresponding author. The data are not publicly available due to ethical restrictions.
